# Generalized Adaptive Diversity Gradient Descent Bit-Flipping with a Finite State Machine

**DOI:** 10.3390/e27010049

**Published:** 2025-01-09

**Authors:** Jovan Milojković, Srdjan Brkić, Predrag Ivaniš, Bane Vasić

**Affiliations:** 1 School of Electrical Engineering, University of Belgrade, 11000 Belgrade, Serbia; mj205018p@student.etf.bg.ac.rs; 2 Tannera Technologies LLC, Veljka Dugosevica 54, 11000 Belgrade, Serbia; srdjan@tannera.io; 3 Department of ECE, University of Arizona, Tucson, AZ 85721, USA; vasic@ece.arizona.edu

**Keywords:** bit-flipping algorithm, gradient descent, iterative decoding, low-density parity-check codes, momentum, finite state machine

## Abstract

In this paper, we introduce a novel gradient descent bit-flipping algorithm with a finite state machine (GDBF-wSM) for iterative decoding of low-density parity-check (LDPC) codes. The algorithm utilizes a finite state machine to update variable node potentials—for each variable node, the corresponding finite state machine adjusts the update value based on whether the node was a candidate for flipping in previous iterations. We also present a learnable framework that can optimize decoder parameters using a database of uncorrectable error patterns. The performance of the proposed algorithm is illustrated for various regular LDPC codes, both in a binary symmetric channel (BSC) and the channel with additive white Gaussian noise (AWGN). The numerical results indicate a performance improvement when comparing our algorithm to previously proposed GDBF-based approaches.

## 1. Introduction

In contemporary communication systems, fast and reliable transmission of information is usually provided by using error correction codes [1]. Low-density parity-check (LDPC) codes [2] can achieve performance close to the Shannon limit, and it is known that the codes can be efficiently decoded, in time linear in their block length [3]. Therefore, these codes have been adopted in recent communication standards: the fifth generation standard for broadband cellular networks (5G NR) uses LDPC codes for the data channels [4], and in digital video broadcasting standards for satellite communications (DVB-S2 and DVB-S2X), these codes are used in combination with Bose–Chaudhuri–Hocquenghem (BCH) codes [5]. They are also a mandatory part of Wi-Fi 6 (IEEE 802.11ax) [6]. In addition, these codes are applied for reliable data storage in solid-state drives (SSDs) [7].

There are various types of decoders for LDPC codes, but the most well known are message-passing (MP) decoders and bit-flipping (BF) decoders. MP decoders operate on a bipartite graph [8], with the structure determined by the parity-check matrix. The bipartite graph consists of two types of graph nodes: variable nodes (VNs) and check nodes (CNs), and a set of edges between them. In the MP paradigm, messages are sent from VNs to the neighbor CNs in one iteration, and vice versa. One of the MP principle’s most well-known algorithms is the Belief Propagation (BP) algorithm [9]. The BP algorithm was introduced on a structure called a tree, but it was later discovered that it can also be used on graphs with cycles. Although the BP decoder has one of the best performances, its hardware implementation is expensive. There are more cost-efficient algorithms that are also in the MP paradigm, with inferior performance when compared to the BP but which are more implementation friendly, such as the min-sum (MS) algorithm [10]. It is well known that BP executes well on codes with long code lengths, while on shorter codes, its performance is lacking. The performance of BP is mostly limited by the presence of trapping sets (TSs), i.e., structures in the bipartite graph that prevent successful decoding. Recently, the Finite Alphabet Iterative Decoders (FAIDs) [11,12] algorithm was proposed to minimize the effect of TSs, which resulted in improved performance in the error floor region.

On the other hand, the original BF algorithm, introduced by Gallager [2], had very poor performance, but it is a fast algorithm with low complexity. In each iteration of this algorithm, the number of unsatisfied parity checks is calculated for every VN, compared to a threshold, and the most critical VNs are flipped. This means that this algorithm is more efficient for implementation, at the price of its performance capabilities. There are several modifications of the BF algorithm that improve performance compared to the original BF algorithm, with a small increase in complexity [13,14]. Wadayama et al. [15] applied the gradient descent method to improve the performance of the bit-flipping algorithm. The resulting algorithm is known as gradient descent bit-flipping (GDBF).

In this paper, we propose a new GDBF algorithm that is effective in the presence of TSs. For short LDPC codes, we show that the concatenation of these decoders outperforms the BP algorithm for a reasonable number of decoding iterations. This effect will be demonstrated for the various lengths of codewords and the most important types of channels.

### 1.1. Related Works

Since the original GDBF algorithm [15] was presented, various modifications and improvements of this algorithm have been proposed. In all of them, a nonlinear energy function is defined for every VN in every iteration based on the received word from the channel, the current codeword estimate, and the number of unsatisfied parity checks for that VN. Only the nodes with the values of the objective function that are higher than the predefined threshold are highlighted and potentially flipped in the corresponding iteration.

In [16], the authors add a new factor to the energy function based on current noise power. This also solves the problem of TSs and introduces a random factor to the algorithm. Another way of introducing randomness into the algorithm is as in [17] where the energy function is not changed (it is only adapted for the binary symmetric channel), but rather, highlighted VNs have some probability of flipping their value. This algorithm is called the probabilistic gradient descent bit-flipping (PGDBF) algorithm.

In [18], information about the previous flipping activity of variable nodes was taken into account in the decision process. Namely, if a VN was flipped in the past iteration, it would not be flipped in the current iteration. In addition, randomness is incorporated into the energy function to highlight the VNs that will be flipped. In [19], the Tabu-List random-penalty GDBF (TRGDBF) algorithm is proposed, where the idea from [18] is combined with a random penalty added to the energy function.

Furthermore, thresholds can be set to provide a flipping of the multiple bits in one iteration. This speeds up the convergence of the algorithm, but there is a possibility that the algorithm may never find a local maximum of the energy function. The information storage bit-flipping (ISBF) [20] combines the basic idea from [19] with adaptive thresholds. In [21], the thresholds change in order of some logic. In [22], the several sets of VNs all have different probability factors for their flipping. In [23], the VNs are grouped into sets depending on their degree, providing the modification suitable for the irregular codes. In [24], energy functions based on previous energy values of the VN are considered. Here, the authors use different weights in the energy function to achieve better results. Different energy functions are also implemented in [25].

In [26], GDBF with momentum (GDBFwM) was introduced, adding a new factor to the energy function. The new factor, named momentum, is used to overcome the problem of TSs by using the previous history of highlighted VNs and removing a predefined chunk of value from their energy function. Moreover, this algorithm supports various values for the threshold that can be used to speed up the convergence. Combining GDBFwM with the basic concept of PGDBF, i.e., flipping the highlighted VNs with some probability, resulted in the PGDBFwM algorithm, also presented in [26]. It has been shown that PGDBFwM has superior performance in the error floor region.

The performance of the GDBF-based algorithm that incorporates the randomness in the decision process can be further improved by using random re-initializations [27]. Combined with further modifications, this approach has the potential to approach the maximum likelihood bound after a large number of iterations [28]. However, the complexity of the probabilistic algorithms is increased as every VN needs an independent random number generator. From the point of hardware implementation, this approach is cost-inefficient.

Therefore, there is a need for an algorithm that could improve the error performance without using any random sequences. The authors in [29] observe that the TSs produce loops in decoding and overcome the problem by using a history of decoded codewords and applying a different algorithm if the loop is detected. Another approach was proposed in [30], where the concept of re-initialization is combined with the information about flipping history, as well as the information about the neighbor nodes. The number of satisfied and unsatisfied checks that connect a suspicious node with other suspicious variable nodes is determined in the first phase. This way, during the course of iteration, such suspicious bits are “distilled” before the final flipping decision. This algorithm is called the Suspicion Distillation Gradient Descent Bit-Flipping (SDGDBF) algorithm.

The idea of concatenating various GDBFwM decoders was proposed in our paper [31]. In the resulting algorithm, named adaptive diversity gradient descent bit-flipping with momentum (AD-GDBFwM), several decoders work in synergy. The value of a restart flag determines whether the input of the next decoder is taken from the output of the previous decoder or the received word from the channel.

In our recent conference paper [32], we showed that the concept from [31] can also be applied to decoding BCH codes. In that paper, we also defined a general concept of potential as the information from the channel that can be used to make more subtle decisions. Basically, the potential is leaned to one side or another regarding highlighting a bit. The resulting algorithm capable of decoding any irregular block code is generalized adaptive diversity gradient descent bit flipping with momentum (gAD-GDBFwM).

### 1.2. Summary and Organization

The solution proposed in this paper contains two components. The first component is a decoding synergy, inspired by [31], where multiple decoders are concatenated in a chain, with the output of one decoder serving as the input for the next. In this way, the decoders with different configuration parameters all work in synergy to produce the decoded codeword. The second component is a variable node potential derived from [32], where potentials are formally introduced concerning BCH codes. In addition, both papers [31,32] incorporate the concept of momentum presented in [26].

The contributions of this study are articulated as follows:

- We propose a finite state machine for updates of potential, which defines which update values should be used based on the previous highlighting activity of the VN. A VN is assumed to be highlighted if its energy function in the current iteration is higher than the predefined threshold. In contrast to the idea presented in Paper [32], the potentials are no longer dependent on iteration but the state of a finite state machine.
- For the proposed algorithm, we present the numerical results for regular LDPC codes, both on the AWGN channel and on the BSC. In [31], the authors presented numerical results for the LDPC codes and the BSC, but the concept of potential was not used in that paper. In [32], numerical results for the simplified algorithm that uses the concept of potential for the BCH codes and the AWGN channel were presented. In that paper, no finite state machine was used to determine the update of potential.
- A new rule that helps in the decoding process of the BSC is introduced. This rule is called the θ rule and it helps the algorithm to overcome the problem related to the formal definition of the sign function. The importance of this rule will be shown later.

The rest of the paper is organized as follows. In Section 2, the system and channel model are presented, and the list of used symbols is given in Table 1. The LDPC codes are formally introduced, and the GDBF algorithm for iterative decoding is also presented in this section. In Section 3, we propose the algorithm that one component decoder runs, a finite state machine for the update of the potential is defined, a typical trapping set for the GDBF algorithm is depicted, and it is shown how GDBF with a finite state machine can correct it. Furthermore, the concatenation of the decoders is presented, and the learnable framework is explained in detail. The numerical results for three regular LDPC codes are presented and discussed in Section 4. In the last section, the concluding remarks are given.

## 2. Preliminaries

Let (n,k) be an LDPC code with a code length *n* and *k* information bits, where R=k/n is the code rate. Let $H_{m\times n}$ be the parity-check matrix of a code. The elements of the matrix H are denoted by $h_{j,i}$, where *j* is the row index, and *i* is the column index. The variable which is connected to the *i*-th column of a parity-check matrix is denoted by $v_{i}$. The parity-check equation related to the *j*-th row of the parity-check matrix is denoted by $c_{j}$. Let $P(v_{i})$ be the set of indices that shows where variable $v_{i}$ is in which check equation, i.e., $P\left(v_{i}\right)=\left\{x|h_{x,i}=1\right\}$. Furthermore, let $Q(c_{j})$ define a set of indices that shows which variables $v_{i}$ are related to the parity-check equation $c_{j}$, that is, $Q\left(c_{j}\right)=\left\{x|h_{j,x}=1\right\}$. With |∗|, let us define the cardinality of a set. The degree of a variable $v_{i}$ is $\left|P(v_{i})\right|$, and the degree of a parity-check equation $c_{j}$ is $\left|Q(c_{j})\right|$. If $|P(v_{i})|=\gamma ,\forall i$ and $|Q(c_{j})|=\rho ,\forall j$, then we have (γ,ρ) as a regular LDPC code. In this paper, we will observe regular LDPC codes.

In this paper, a bipolar representation of the codewords will be used. This means that the encoder output is {−1,+1}, so a transmitted codeword is $x=(x_{1},x_{2},\ldots ,x_{N})$ where $x_{i}\in \left\{\pm 1\right\}$. In this paper, we will analyze the transmission through two types of channels:

- If the AWGN channel is used, at the output of the channel the vector $y=(y_{1},y_{2},\ldots ,y_{N})$, $y\in R^{N}$ is received. The channel quality is determined by the received signal-to-noise ratio (SNR).
- If the BSC is used, at the output of the channel the vector $y=(y_{1},y_{2},\ldots ,y_{N})$, where $y_{i}\in \left\{\pm 1\right\}$. This can be considered as the special case of the AWGN channel, where a hard decision is applied prior to the decoding. The channel quality is determined by the crossover probability, denoted by α.

The original GDBF algorithm, as defined in [15], steams to maximize the objective function, defined as

(1) $$\begin{array}{c} f\left(\hat{x}\right)=\sum_{i=1}^{N}{\hat{x}}_{i}y_{i}+\sum_{j=1}^{M}\prod_{i\in Q(c_{j})}{\hat{x}}_{i}, \end{array}$$

where $\hat{x}$ denotes the estimated codeword in the decoder. In the above expression, the first term represents the correlation between the received codeword and the potential solution, while the second term represents how many check equations are satisfied. The second term has its maximum value only if all the check equations are satisfied and have a penalty factor in Equation (1).

All BF-based iterative decoding algorithms use the same framework, i.e., they calculate the local energies of all variables with the inversion function in every particular iteration. The variables with minimum energy are highlighted, and some of the highlighted variables are flipped to create the updated estimation of the codeword. The decoding is terminated if all parity checks are satisfied, or if the maximum allowed number of iterations is reached.

The inversion function for the GDBF algorithm in the *l*-th iteration is defined as [15]

(2) $$\begin{array}{c} E_{i}^{\left(\ell \right)}=y_{i}{\hat{x}}_{i}^{\left(\ell -1\right)}+\sum_{j\in P(v_{i})}\prod_{o\in Q(c_{j})}{\hat{x}}_{o}^{\left(\ell -1\right)}. \end{array}$$

The improvements of the GDBF algorithms were mostly related to the method selection of the highlighted variables, which should be flipped in a certain iteration. In the PGDBF algorithm, proposed in [17], the variable bits that should be flipped are chosen randomly from the set of highlighted variables. In the TRGDBF algorithm, the variables flipped in the previous iteration are not flipped, although they belong to the set of highlighted variables. The GDBFwM algorithm extends the effect of memory in variable nodes, and the variables that were flipped in the recent iteration are unstimulated to be flipped in the following iterations. The other recent papers combined these approaches with re-initializations and concatenation of the component decoders [27,30,31].

## 3. Framework

We assume that full decoder gAD-GDBFwM-wSM consists of *T* decoders in the chain, and the maximum allowed number of iterations for the decoding is $L_{max}=\sum_{t=1...T}L_{max,t}$, where $L_{max,t}$ denotes the number of iterations of the *t*-th decoder that will run the algorithm. Based on the flag restart value of the *t*-th decoder, denoted as $r_{flag}^{\left(t\right)}$, the inputs of the corresponding component decoder will be determined.

In the next subsections, the algorithm applied to every component decoder will be explained, the method of concatenation of the component decoders will be described, and the applied learning method for optimizing the decoder parameters will be presented.

### 3.1. Description of One Component Decoder

In a component decoder, the energy function for the *i*-th variable bit in the *l*-th iteration is defined as

(3) $$\begin{array}{c} E_{i}^{\left(\ell \right)}=w_{1}y_{i}{\hat{x}}_{i}^{\left(\ell -1\right)}+w_{2}\sum_{j\in P(v_{i})}\prod_{o\in Q(c_{j})}{\hat{x}}_{o}^{\left(\ell -1\right)}+m_{\mu_{i}}. \end{array}$$

where $w_{1}$ and $w_{2}$ are learnable weights that have the same value for all variable bits (for the regular codes; there is no need to specify separate values for various variables, as shown in [31]).

Factor $m_{\mu_{i}}$ represents the momentum of the decoder, where $\mu_{i}$ represents the momentum state of the *i*-th variable. Momentum can take any value from positive integers, that is, $m_{\mu_{i}}\in \left\{0,1,\ldots ,I\right\}$, $I\in N^{+}$, and the momentum vector looks like $m=(m_{1},m_{2},\ldots ,m_{L^{\prime }},0)$, where $m_{1}\geq m_{2}\geq m_{3}\ldots \geq m_{L^{\prime }}\geq 0$. The value for $\mu_{i}$ is calculated based on the past highlighting activity of the variable $v_{i}$; if the last time a variable $v_{i}$ was highlighted at *q*-th iteration, then

(4) $$\begin{array}{c} \mu_{i}=\min\left(\ell -q,L^{\prime }+1\right). \end{array}$$

Let $\mu =(\mu_{i})$, where μ is a vector of all values of $\mu_{i}$. We will refer to μ as the state of momentum.

The set of highlighted variables is given as

(5) $$\begin{array}{c} F^{\left(\ell \right)}=\left\{v_{i}|E_{i}^{\left(\ell \right)}\leq E_{\min}^{\left(\ell \right)}+\delta \right\}, \end{array}$$

where

(6) $$\begin{array}{c} E_{\min}^{\left(\ell \right)}=\min_{i=1,2...n}\left(E_{i}^{\left(\ell \right)}\right), \end{array}$$

denotes minimum energy in the *ℓ*-th iteration, and δ≥0 represents the margin (threshold) that enables the highlighting of multiple bits.

Now, the potential of the variable $v_{i}$ is updated with the following rule

(7) $$\begin{array}{c} r_{i}^{\left(\ell +1\right)}=r_{i}^{\left(\ell \right)}+\mathrm{sign}\left(r_{i}^{\left(\ell \right)}\right)*s_{v_{i}}, \end{array}$$

where $s_{v_{i}}$ is the updating value that corresponds to the state of the variable $v_{i}$, as defined in the next subsection. Here, $r_{i}^{\left(\ell \right)}$ represents the value of the potential for the *i*-th variable in the *ℓ*-th iteration.

Note that in Equation (7), especially in the case where the BSC is used, the potential may be equal to zero, that is, $r_{i}^{\left(\ell +1\right)}=0$. In such a case, a θ rule should be applied. A θ rule is a rule in which if the potential was pushed from one side to another, and its potential is equal to zero, then we add θ, a small number, to the side we are already pushing.

Formally speaking, θ rule can be represented as

(8) $$\begin{array}{c} \left(r_{i}^{\left(\ell +1\right)}=0\right)\Rightarrow \left(r_{i}^{\left(\ell +1\right)}=-\mathrm{sign}\left(r_{i}^{\left(\ell \right)}\right)\theta \right). \end{array}$$

After the potential is updated, the estimated codeword for the next iteration can be updated as

(9) $$\begin{array}{c} {\hat{x}}_{i}^{\left(\ell +1\right)}=\mathrm{sign}\left(r_{i}^{\left(\ell +1\right)}\right), \end{array}$$

where ${\hat{x}}_{i}^{\left(\ell \right)}$ represents the hard decision of the *i*-th bit of the codeword for the *ℓ*-th iteration, while $r_{i}^{\left(\ell \right)}$ represents the potential (i.e., soft decision of the codeword). It can be seen here that even though a variable is highlighted, its potential will change, but its hard decision may stay the same.

Therefore, we can summarize that the input parameters of the *t*-th component decoder are the received codeword y, the initial vector of potentials $r_{in}^{\left(t\right)}$, the momentum states $\mu_{in}^{\left(t\right)}$, and the potential states $P_{in}^{\left(t\right)}$. The finite state machine is used to update the potential states $P_{in}^{\left(t\right)}$, as will be described in the next subsection. At this point, we can present an algorithm that a single decoder executes (specified in Algorithm 1), as well as a method for concatenation of decoders.
**Algorithm 1** gAD-GDBFwM with Finite State Machine in one component decoder**Input:**  $y=\left(y_{i},y_{2},\ldots ,y_{N}\right)\in R^{N}$,   $r_{in}^{\left(t\right)}$,   $\mu_{in}^{\left(t\right)}$,   $P_{in}^{\left(t\right)}$,   δ
**Output:**  $\hat{x}=\left({\hat{x}}_{1}^{\left(\ell \right)},{\hat{x}}_{2}^{\left(\ell \right)},\ldots ,{\hat{x}}_{N}^{\left(\ell \right)}\right)\in {\left\{\pm 1\right\}}^{N}$,   $r_{out}^{\left(t\right)}$,   $\mu_{out}^{\left(t\right)}$,   $P_{out}^{\left(t\right)}$
**Initialization:**  ℓ=1, $r^{\left(1\right)}=r_{in}^{\left(t\right)}$, $\mu =\mu_{in}^{\left(t\right)}$, $P=P_{in}^{\left(t\right)}$
${\hat{x}}_{i}^{\left(1\right)}=\mathrm{sign}\left(r_{i}^{\left(1\right)}\right)$, i=1,2,…,N $s_{j}^{\left(1\right)}=\prod_{i\in Q(c_{j})}{\hat{x}}_{i}^{\left(1\right)}$, j=1,2,…,M **while** ($\ell \leq L_{\max,t})$ or ($s_{j}^{\left(\ell \right)}=1,\forall j$) **do**
   $E_{\min}^{\left(\ell \right)}=+\infty$
   **for** i=1,…,N **do**
     $E_{i}^{\left(\ell \right)}=w_{1}^{\left(\ell \right)}y_{i}{\hat{x}}_{i}^{\left(\ell \right)}+w_{2}^{\left(\ell \right)}\sum_{j\in P(v_{i})}s_{j}^{\left(\ell \right)}+m_{\mu_{i}}$
     $E_{\min}^{\left(\ell \right)}=\min\left\{E_{\min}^{\left(\ell \right)},E_{i}^{\left(\ell \right)}\right\}$
   **end for**
   $F^{\left(\ell \right)}=\left\{v_{i}|E_{i}\leq E_{\min}+\delta \right\}$
    update μ accordingly to $F^{\left(\ell \right)}$
    update P accordingly to $F^{\left(\ell \right)}$ and state machine
   $r_{i}^{\left(\ell +1\right)}=r_{i}^{\left(\ell \right)}+s_{v_{i}}\mathrm{sign}\left(r_{i}^{\left(\ell \right)}\right)$, i=1,2,…,N
    apply rule θ if necessary and update $r_{i}^{\left(\ell +1\right)}$, i=1,2,…,N
   ${\hat{x}}_{i}^{\left(\ell +1\right)}=\mathrm{sign}\left(r_{i}^{\left(\ell +1\right)}\right)$, i=1,2,…,N
   $s_{j}^{\left(\ell +1\right)}=\prod_{i\in Q(c_{j})}{\hat{x}}_{i}^{\left(\ell +1\right)}$, j=1,2,…,M
   ℓ=ℓ+1
**end while**
$r_{out}^{\left(t\right)}=r^{\left(\ell \right)}$, $\mu_{out}^{\left(t\right)}=\mu$, $P_{out}^{\left(t\right)}=P$


### 3.2. Description of the Finite State Machine

We now introduce the finite state machine S that will be used to update the potentials. Each state *S* in S consists of an update value that corresponds to the state, denoted by *s*. Among |S| states, there are three subsets of states: plus states $S_{+}=\left\{S_{+,1},S_{+,2}\ldots S_{+,\eta }\right\}$, minus states $S_{-}=\left\{S_{-,1},S_{-,2}\ldots S_{-,\zeta }\right\}$, and there is a neutral state $S_{0}$. The subset $S_{+}$ has all update values $s_{+}\geq 0$; the values in states $S_{-}$ have update values of $s_{-}<0$ and $s_{0}=0$. The starting state of a decoder, denoted by $S_{start}$, can be any state in S.

**Figure 1:**
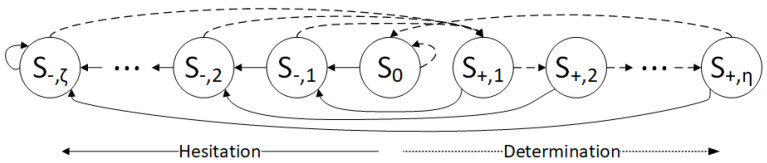
Finite state machine used to update the potentials. The circles represent the states, while the arrows represent a transition between them. The full lines represent transitions when the variable is highlighted, while the dashed lines represent transitions when the variable is not highlighted.

The transitions between states are triggered by the event of highlighting the variables. When the variable is highlighted, it is a candidate for flipping and its potential should be pushed toward the opposite direction than its current value of potential, which means that some state of $S_{-}$ should be chosen during the update procedure. If the variable is not highlighted, then some state from the set $\left\{S_{+}\cup S_{0}\right\}$ should be chosen. If η=0, then if the variable is not highlighted, it should go to the state $S_{0}$. Here, it should be noted that ζ>0 because if ζ=0, the variable will never be flipped. If the variable in $S_{0}$ is highlighted, then it should go to a state in set $S_{-}$ and if not, it remains in the state $S_{0}$. In Figure 1, the full line represents the transition between states when the variable is highlighted, and the dashed lines represent the transition when the variable is not highlighted.

In Figure 1, the more the variable goes to the left states, the more uncertain that variable is. Therefore, moving in the direction from $S_{0}$ towards $S_{-,\zeta }$ corresponds to a Hesitation phase, where uncertainty gets higher. In contrast to this concept, there is a direction of Determination, where certainty gets higher. If the variable is not highlighted, it is not a candidate for flipping in that iteration, and its potential value should remain the same sign. If the variable is not highlighted in a few subsequent iterations, the more certain the variable is, the more its potential needs to be pushed in the same direction. After η consecutive iterations without highlighting, the variable enters the state $S_{0}$ where no further updates are necessary until the next highlighting of the variable. The transitions between states can be configured differently, but it is important to follow the general rules given above. As explained in Algorithm 1, the variable should be flipped at the moment when the sign of its potential is changed.

### 3.3. Finite State Machine and Trapping Set Example

**Figure 2:**
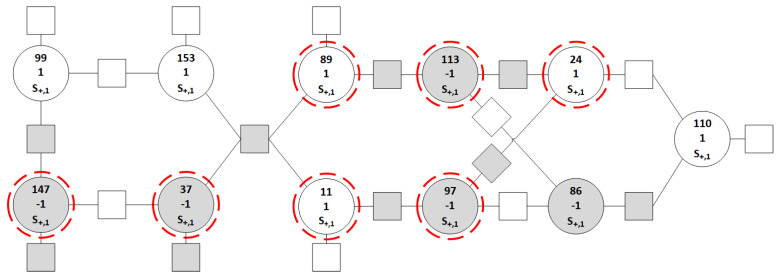
An example of a trapping set. Here the circles represent the variable nodes. The first number in the circle represents the VN number, the second number represents the value of the potential, and, finally, the state for that variable is presented.

In this section, it will be shown how an error pattern that is uncorrectable by using GDBF can be corrected by using GDBF with the finite state machine. This TS, obtained at the output of the BSC, is given in Figure 2. The correct VNs are represented with white circles, while erroneous VNs are represented with gray circles. Satisfied parity checks are represented by white squares, while unsatisfied parity checks are represented by using gray squares.

In the first iteration, the GDBF algorithm flips all highlighted VNs ($v_{11}$, $v_{24}$, $v_{37}$, $v_{89}$, $v_{97}$, $v_{113}$, and $v_{147}$). In the second iteration, the GDBF algorithm will highlight $v_{24}$, $v_{97}$, and $v_{113}$. After those variable bits are flipped in the GDBF algorithm, in the third iteration, the GDBF algorithm highlights $v_{11}$, $v_{24}$, and $v_{89}$ and flips them. After this iteration, the decoding problem will occur; that is, the GDBF algorithm will now only flip nodes $v_{24}$ and $v_{110}$ in each subsequent iteration and will never stop flipping these variable nodes.

**Figure 3:**
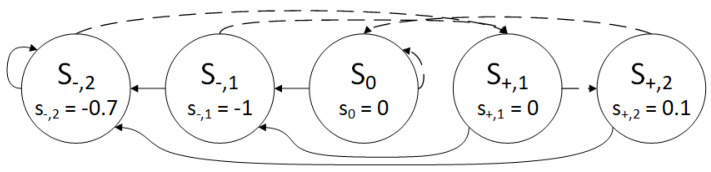
The finite state machine for the example used in Figure 2.

Therefore, the GDBF algorithm with a finite state machine and with learnable weights will be used. It will be shown that only the finite state machine with learnable weights $w_{1}$ and $w_{2}$ without momentum can correct the given TS. In the case of the BSC, the received word is $y=(y_{1},y_{2},\ldots ,y_{N})$, where $y_{i}\in \left\{\pm 1\right\}$. The initial value of the potential is $r_{i}=y_{i}$. In this example, we assume the values $w_{1}=w_{2}=3.2$, and the finite state machine has |S|=5 states: the neutral state $S_{0}$, η=2 plus states $S_{+}=\left\{S_{+,1},S_{+,2}\right\}$, and ζ=2 minus states $S_{-}=\left\{S_{-,1},S_{-,2}\right\}$, as illustrated in Figure 3. It is assumed that the starting state for the finite state machine is $S_{+,1}$, and all variable nodes will be in that state with potential $r_{i}^{\left(1\right)}=1$ at the start of the decoding process. In Figure 2, for every variable node, we wrote: the VN number, the current state, and the corresponding value of the potential.

As the decoded codeword ${\hat{x}}_{i}^{\left(1\right)}=\mathrm{sign}\left(r_{i}^{\left(1\right)}\right)$ has unsatisfied checks, the potentials of the variables are updated according to the finite state machine.

**Figure 4:**
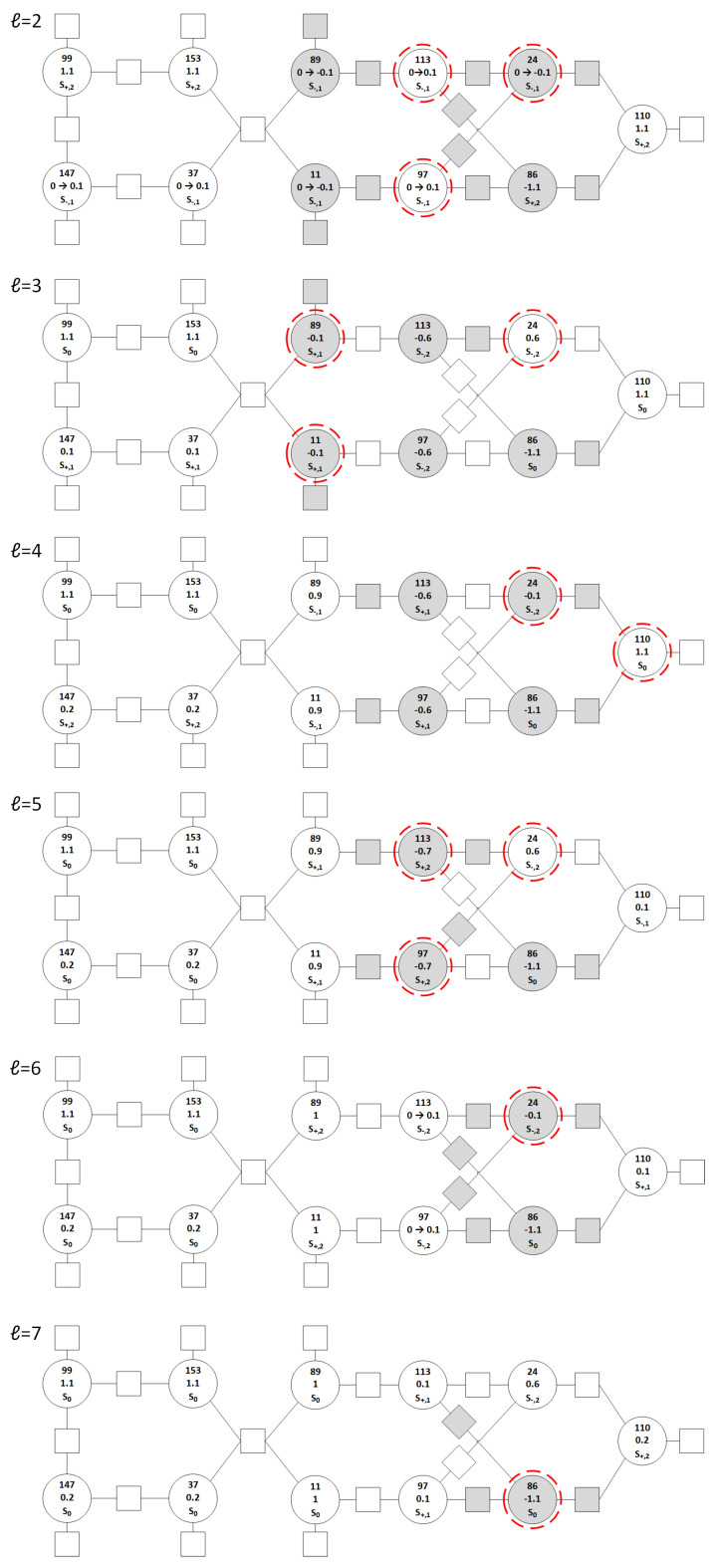
Trapping set analysis with the finite state machine. The *ℓ* represents the current iteration, and the parameters inside VNs represent the parameters at the beginning of that iteration.

- If the variable is not highlighted in the first iteration, it will reach the state $S_{+,2}$, according to the diagram shown in Figure 3 following the dashed lines. The updated potentials for these VNs are $r_{86}^{\left(2\right)}=-1.1$, $r_{99}^{\left(2\right)}=1.1$, $r_{110}^{\left(2\right)}=1.1$, and $r_{153}^{\left(2\right)}=1.1$.
- If the VN is highlighted in the first decoding iteration, then it will go from the state $S_{+,1}$ to the state $S_{-,1}$, according to the diagram shown in Figure 3 following the full lines. The corresponding update is $-1\mathrm{sign}(r_{i}^{\left(1\right)})$, and therefore, $r_{i}^{\left(2\right)}=0$ for all highlighted VNs. In this case, the θ rule has to be applied to enable a correct codeword estimation. As an example, if the potential for the variable node $v_{113}$ after the update would be equal to $r_{113}^{\left(2\right)}=0$, it is not clear how to calculate ${\hat{x}}_{113}^{\left(2\right)}$. When this scenario occurs, the parameter θ is used to push it a bit more to the opposite side when compared to the potential from the previous iteration, and the potential will be equal to $r_{113}^{\left(1\right)}=+\theta$. The modification according to the θ rule is denoted as 0→±θ in Figure 4. We set the value θ=0.1 in this example, and therefore $r_{11}^{\left(2\right)}=-0.1$, $r_{24}^{\left(2\right)}=-0.1$, $r_{37}^{\left(2\right)}=0.1$, $r_{89}^{\left(2\right)}=-0.1$, $r_{97}^{\left(2\right)}=0.1$, $r_{113}^{\left(2\right)}=0.1$, and $r_{147}^{\left(2\right)}=0.1$.

After the first iteration, all highlighted bits are flipped, and the estimated codeword is presented in Figure 4, for ℓ=2. We emphasize that the same codeword estimation is obtained for the case when the original GDBF algorithm is applied. However, in our algorithm, the real-valued potential is assigned for every VN, and it can be used to obtain more reliable decisions in the next iterations.

In the second iteration, the highlighted VNs are $v_{24}$, $v_{97}$, and $v_{113}$, and Equation (7) was applied to all VNs to update the corresponding values of potential. The VNs that are not highlighted in this iteration can be divided into two groups: one that reaches the state $S_{+,1}$ and the other group of VNs that reaches the state $S_{0}$, but in both cases, there is no update of the potential. The next state for all highlighted VNs will be $S_{-,2}$, with the update of potential equal to $s_{-,2}=-0.7$. Therefore, we finally obtain $r_{24}^{\left(3\right)}=-0.6$, $r_{97}^{\left(3\right)}=-0.6$, and $r_{113}^{\left(3\right)}=-0.6$, and these bits are flipped again in the second iteration. The same VNs would be flipped in the GDBF algorithm.

In ℓ=3, the algorithm highlights VNs $v_{11}$, $v_{24}$, and $v_{89}$. Two highlighted variables which were in state $S_{+,1}$ will go to state $S_{-,1}$. On the other hand, the variable $v_{24}$ that was in state $S_{-,2}$ will remain in that state. It can be seen that all the VNs that are highlighted will be flipped, as their potential will change the sign. Although the same flipping decision would be made in the GDBF algorithm, the potential for $v_{24}$ indicates that the reliability of this decision is not so high.

For iteration ℓ=4, VNs $v_{24}$ and $v_{110}$ are highlighted. Now, since $v_{110}$ was in the state $S_{0}$ it will go to the state $S_{-,1}$ which has the update value −1. The updated potential $r_{110}^{\left(5\right)}=0.1$ will not change sign, i.e., it will not be flipped. On the other hand, when looking at the GDBF algorithm all those highlighted VNs will be flipped. In the algorithm we proposed in this paper, the finite state machine slows down the flipping decisions. As a result, even if this pattern is uncorrectable by using GDBF, it does not represent the trapping set for the GDBF algorithm with the finite state machine.

For the iteration ℓ=5, the difference when compared to GDBF is visible. VN $v_{110}$ is no longer highlighted, and its potential remains the same as in the previous iteration. Now, the highlighted VNs are $v_{24}$, $v_{97}$, and $v_{113}$. Since VNs $v_{97}$ and $v_{113}$ were in state $S_{+,2}$ and highlighted, they will go to state $S_{-,2}$, and for them, the θ is applied and they will be flipped. The VN $v_{24}$ remains in the state $S_{-,2}$ and will also be flipped.

Looking at the iteration ℓ=6, now only two VNs are erroneous, $v_{24}$ and $v_{86}$. Firstly, $v_{24}$ will be highlighted because its energy function has the first parameter equal to $w_{1}*\left(-1\right)$ (see Equation (3)), which is added to the number of unsatisfied parity checks. This variable $v_{24}$ remains in the same state $S_{-,2}$ and will be flipped.

In the next iteration (ℓ=7), only one VN is in an incorrect state, and that VN is $v_{86}$. Since that VN is highlighted, it will first go to state $S_{-,1}$ where its potential will be updated by value −1 and its potential will be $r_{86}^{\left(8\right)}-0.1$ at the beginning of the eight iteration. Although this is an erroneous VN and is highlighted, it will not be flipped in this iteration, as the sign of the corresponding potential is unchanged. In the eight iteration, the same variable node $v_{86}$ is highlighted again. Therefore, it will reach the state $S_{-,2}$ and will be flipped.

Here, it took eight iterations for the GDBF algorithm with the presented finite state machine (without momentum) to correct this TS of GDBF. The most crucial iteration is the fourth iteration in which VN $v_{110}$ is not flipped, but rather its potential is leaned to one side.

In the previous example, it has been shown that the highlighted VN will not be flipped in some situations. Therefore, the GDBF algorithm that uses a finite state machine to update the potentials can slightly slow down the decoding process. However, the numerical results presented in the next section indicate that the benefits of this approach justify this minor drawback.

It can be noticed that the GDBF-based algorithms that incorporate randomness in the decision process can result in a similar effect. For example, in the PGDBF algorithm with parameter p=0.7 [17], in every particular iteration, about 70 percent of the highlighted VNs are randomly selected for the flipping. In our algorithm, we avoid random selection and there is no need for the implementation of the multiple independent random sequences in the variable nodes (that is known as a complex task). On the contrary, we use the deterministic finite state machine to obtain the soft information that helps us obtain a reliable decision.

### 3.4. Concatenation of the Component Decoders

Here, we discuss how to connect the component decoders, and the corresponding inputs and outputs will be defined. These parameters mostly depend on the position in the chain, denoted by *t*, and the value of the corresponding restart flag $r_{flag}^{\left(t\right)}$, as follows:

- For the first decoder (t=1), the input vector of the potentials is equal to the received word, i.e., $r_{in}^{\left(1\right)}=y$. Furthermore, momentum states and potential states are equal for all variables, $\mu_{in,i}^{\left(t\right)}=L^{\prime }+1,i=1,2,\ldots ,N$, and $P_{in,i}^{\left(t\right)}=S_{start}^{\left(t\right)},i=1,2,\ldots ,N$.
- If t>1 and $r_{flag}^{\left(t\right)}=1$, the inputs are also defined with the expressions: $r_{in}^{\left(t\right)}=y$, $\mu_{in,i}^{\left(t\right)}=L^{\prime }+1,i=1,2,\ldots ,N$, and $P_{in,i}^{\left(t\right)}=S_{start}^{\left(t\right)},i=1,2,\ldots ,N$.
- If t>1 and $r_{flag}^{\left(t\right)}=0$, the input vector of the potentials is equal to the output vector of potentials from the previous decoder: $r_{in}^{\left(t\right)}=r_{out}^{\left(t-1\right)}$, and using the similar approach $\mu_{in}^{\left(t\right)}=\mu_{out}^{\left(t-1\right)}$, and $P_{in}^{\left(t\right)}=P_{out}^{\left(t-1\right)}$.

It can be noticed that the momentum state $\mu^{\left(t\right)}$ can be longer than the momentum state for the previous decoder $\mu^{\left(t-1\right)}$. In such a case, the zeros can be added to expand the momentum vector of the *t*-th decoder. Moreover, the decoders do not need to have the same number of states, and the transitions between the states of the decoders can be defined. A function $S_{t}:S_{t-1}\to S_{t}$ can be defined to provide the mapping of the states of the (t−1)-th decoder to the *t*-th decoder. This function can be used to connect vectors $P_{in}^{\left(t\right)}$ into $P_{out}^{\left(t\right)}$, even in the case when corresponding finite state machines do not have an equal number of states.

### 3.5. Learnable Framework

**Figure 5:**
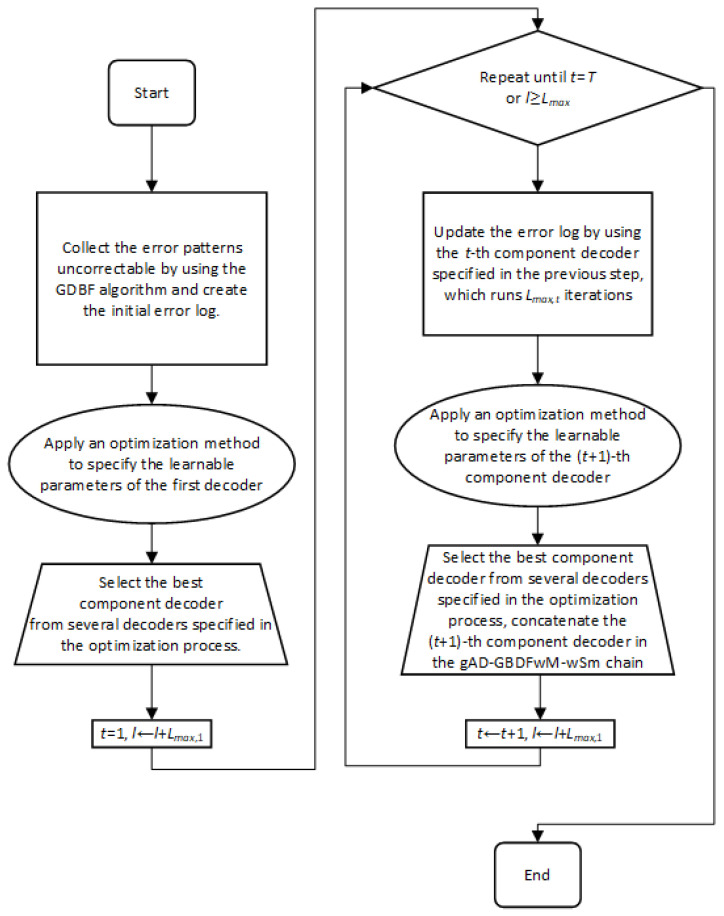
Process for learnable framework.

In this section, the learnable framework used to obtain all parameters for the decoders will be described. The process of the learnable framework can be seen in Figure 5.

In the first step of the optimization process, the Monte Carlo simulation method is used to acquire a set of error patterns that cannot be corrected by using the original GDBF decoder (as defined in [15]). The simulation must be run for the channel conditions that correspond to the error floor region (a small crossover probability α in the BSC or a high SNR in the AWGN channel), as the error patterns in this region are mostly uncorrectable due to the trapping sets. Every transmitted codeword should be decoded for a large number of iterations, and the number of sent codewords is adjusted to collect a few thousand uncorrectable error patterns. The resulting database set of uncorrectable error patterns is denoted as the initial error log.

In the next step, the learnable parameters of the first component decoders have to be optimized using an optimization algorithm based on the initial error log. As most optimization algorithms are random by nature and depend on the starting point in the optimization process, the algorithm may be run for different speeds to improve the optimization efficiency. In such a case, the decoder that corrects the largest number of error patterns in the initial error log is selected as the first component decoder.

In the third step of the learnable procedure, all error patterns correctable by using the chosen decoder during $L_{max,1}$ have to be removed from the initial error log. As the optimization accuracy depends on the size of the error log, new error patterns that are uncorrectable by the first component decoder should be added. Therefore, the first step of this procedure will be repeated to acquire additional error patterns that cannot be corrected by the first component decoder, obtained in the previous step. Finally, we update the error by merging the remaining error patterns from the initial error log (still uncorrectable by using the first decoder in the chain) with the new error patterns acquired in this step.

Using the updated error log, the same optimization algorithm that is used in the second step is applied to optimize the parameters of the next component decoder. Using the rules defined in the previous subsection, this component decoder is added to the chain.

The two previous steps are repeated until the required number of component decoders is reached (where the *t*-th component decoder is optimized based on the error log updated by using the (t−1)-th decoder), or until the maximum allowed number of iterations is reached. The decoders are learned one by one, which can also be seen from the procedure presented in Figure 5.

In this paper, the genetic algorithm (GA) is used to optimize the learnable parameters of every particular decoder. The GA is a population-based algorithm, where every problem solution is represented by a chromosome [33]. Each problem solution has its fitness value, and the higher fitness values correspond to better optimization. In the first generation, the chromosomes are chosen randomly and then the individuals are chosen in a selection phase. The best individuals are transferred to the next generation (elitism). The chosen individuals begin a combination process in which their genes are combined to generate a new solution to the problem (combination), and after the mutation phase, a new population is created. After some number of generations, we terminate the algorithm and use the best chromosome as the solution to the given problem.

In order to use some machine-type optimization procedures, there is a need to quantize all of the learnable parameters. This means that they are in some range and can have some values only from a set of discrete values. Then, we can represent those values with bits, append all bits together, and use a genetic algorithm to obtain a set of parameters for one decoder.

In our optimization problem, there are three types of decoder parameters as follows:

- The parameters which are heuristically chosen for the *t*-th component decoder, such as the starting state $S_{start}^{\left(t\right)}$, the maximum number of iterations $L_{max,t}$, and the margin δ. These are parameters which are known before the machine learning optimization procedure.
- The basic parameters of the *t*-th decoder are the number of states for the finite state machine $S_{t}$, the length of the momentum vector $L^{\prime }$, the maximum value which momentum can take *I*, and the value of the flag restart $r_{flag}^{t}$. For one combination of these parameters, one GA optimization has to be run.
- The parameters obtained as a result of the GA optimization are learnable weights $w_{1}$ and $w_{2}$, state values *s* for states in the finite state machine, and momentum vector m.

The GA optimization gives us some parameters for $w_{1}$, $w_{2}$, *s*, and m, for one combination of the basic parameters. The efficiency of optimization is measured with the achieved fitness rate (FR), which corresponds to the percentage of corrected error patterns in the error log used for the learning of the GA. Therefore, the highest GA value is achieved for the optimal combination of the basic parameters. This way, the optimal combination of parameters $S_{t}$, $L^{\prime }$, *I*, and $r_{flag}^{t}$ can also be determined.

## 4. Numerical Results

In this section, we will compare the performance of the gAD-GDBFwM-wSM algorithm with the previously analyzed algorithms. Frame error rate (FER) after the decoding is estimated by comparing the transmitted codewords and the estimated codewords at the output of the decoder, by using the Monte Carlo simulation process [34]. The numerical results are presented for the case when the number of transmitted codewords is adjusted to detect at least 100 errors. The maximum number of iterations for the analyzed iterative algorithms is set to $L_{max}=300$, if it is not specified otherwise.

The starting state $S_{start}=S_{+,1}$. If η=0, then $S_{start}=S_{0}$. The starting position for the momentum, when restart is active, should always be $L^{\prime }+1$, i.e., we chose m$(L^{\prime }+1)=0$. Regarding the learning procedure and the momentum vector, several sets of momentum vectors are used for GA optimization. One set of momentum vectors is used in one GA optimization. In Table 2, we give the parameters of momentum sets used in the GA optimization.

The results that will be shown are for three codes: Tanner (155, 64) code, i-RISC (1296, 648) code, and IEEE 802.3an (2048,1720) code. Details about these codes are given in the following sections. FER performance was analyzed for the BSC and AWGN channels. For each code and each channel, we will give a detailed description of the methods and parameters used throughout the procedure for producing the gAD-GDBFwM-wSM decoder. Furthermore, the optimized parameters of gAD-GDBFwM-wSM in all analyzed scenarios are given online in the address specified in [35].

### 4.1. Tanner Code

First, we present the numerical results for the Tanner (155,64) code with the code rate R=0.4129, for the case where the BSC is used for transmission. This code is regular with variable node degree γ=3, the degree of parity-check equations ρ=5, the minimum Hamming distance $d_{min}=20$, and the construction method described in [36]. This code will be called the Tanner code in the rest of the paper.

**Figure 6:**
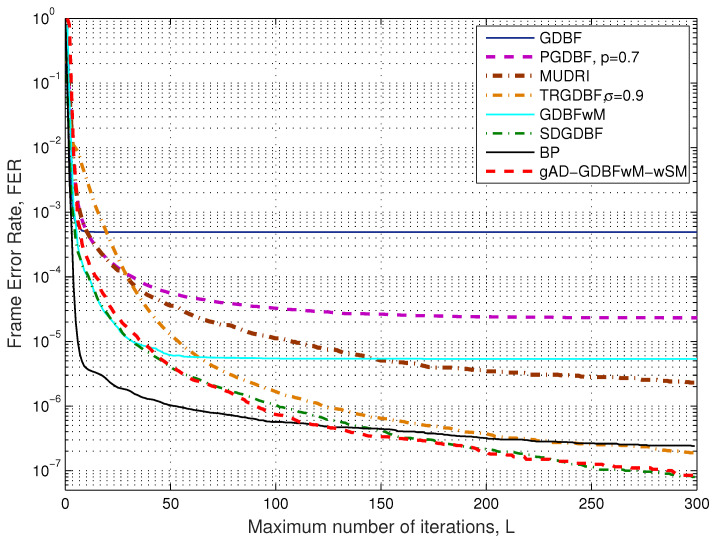
Performance as a function of $L_{max}$, BSC, Tanner code (155, 64), γ=3 and ρ=5.

The FER as a function of parameter $L_{max}$ for the Tanner code is presented in Figure 6, for the fixed BSC crossover probability α=0.01. As shown in [26], the incorporation of momentum in the energy function improves the performance of the GDBF decoder. Therefore, GDBF is far inferior to GDBFwM (for any value of $L_{max}$). If statistically independent random sequences are incorporated in every VN, as proposed in PGDBF, the FER can be highly reduced after a large number of iterations [17,27,28]. The convergence speed can be improved if a binary random sequence is added in the energy function, and the VNs flipped in the previous iteration are prevented from flipping in the current iteration, as in the tabu-list random GDBF (TRGDBF) [19]. The FER performance can be further improved using a complex but completely deterministic algorithm proposed in [30]. The performance of the gAD-GDBFwM-wSM algorithm proposed in this paper is presented for the case where the momentum vector and the decoder coefficients are optimized to achieve the minimum FER. It is assumed that every component decoder has to complete decoding after $L_{max,t}=30$ iterations, and every decoder can take momentum values from the predefined momentum sets ($MS_{1},MS_{2},MS_{3},MS_{4}$).

In the optimization process, four combinations of basic parameters were investigated, as it is assumed that the finite state machine has parameters like ${SM}_{1}$ and ${SM}_{2}$ from Table 3, and two flag restart values were considered ($r_{flag}=0$, $r_{flag}=1$). For every particular combination of the basic parameters, the optimization of the momentum vector and the decoder coefficients is performed by using the GA, where the optimization is performed with 40 chromosomes in 40 generations. The combination with the highest fitness value is chosen as the optimal, and the chosen momentum and coefficients completely define that component decoder. The procedure is repeated according to Figure 5 to specify the optimal parameters of all component decoders. The corresponding numerical results are also presented in Figure 6. It is obvious that the gAD-GDBFwM-wSM algorithm achieves much lower FER values compared to the GDBF algorithm when α=0.01. In addition, it outperforms all analyzed probabilistic algorithms (the PGDBF, the MUDRI, and the TRGDBF) for any value of $L_{max}$.

The gAD-GDBFwM-wSM algorithm is based on the concatenation of optimized component GDBFwM-based decoders. It is obvious that this concatenation approach prevents the saturation that is visible for the single GDBFwM decoder (that does not reduce FER after 50 iterations, as shown in Figure 6. However, it can be noticed that a single GDBFwM decoder for certain values of parameters $L_{max}$ outperforms the gAD-GDBFwM-wSM algorithm. This surprising effect can be easily explained if we observe that every component decoder is optimized to minimize FER at the output of the *t*-th decoder after $L_{max,t}$ iterations. If $L_{max,t}=30$ for any value of *i*, this decoder is forced to be optimal after 30, and 60, iterations, and there is no guarantee that it will be optimal for $L_{max}=45$. This side effect can be mitigated if the value for $L_{max,t}$ is further reduced for the first decoders in the chain, or the optimization criterion is slightly changed.

The proposed algorithm has performance comparable to that of the SDGDBF algorithm. It should be noted that the optimization procedure for gAD-GDBFwM-wSM, does not require the additional hardware overhead as in the SDGDBF (as explained in Section 4.2 in [30]). In our framework, the optimization process of the gAD-GDBFwM-wSM decoder parameters can be performed offline. Therefore, the same hardware is used in every component decoder, while the adaptation of the momentum values and the decoder coefficients are performed by their refreshment after $L_{max,t}$ iterations.

**Figure 7:**
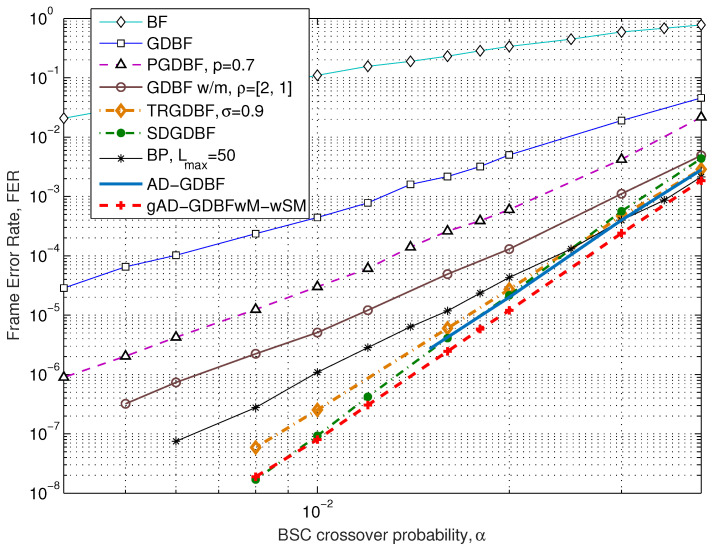
Performance of the various decoders, BSC, Tanner code (155, 64), γ=3 and ρ=5.

In Figure 7, we present FER as a function of α, where the maximum number of iterations is $L_{max}=300$ if not specified otherwise. It is obvious that the gAD-GDBFwM-wSM algorithm’s performance is far superior when compared to the BF, the GDBF, the PGDBF, and the GDBFwM algorithms. In the error floor region, the proposed algorithm also outperforms the TRGDBF algorithm and the state-of-the-art BP algorithm. For the analyzed range of the parameter α, the gAD-GDBFwM-wSM decoding algorithm has a similar performance as the recently proposed SDGDBF algorithm.

### 4.2. i-RISC Code

Next, we compare the FER performance of the proposed algorithm with the other GDBF-based algorithms for the (1296, 648) QC-LDPC code with code rate R=0.5. The construction method for this regular code is presented in [37]. This code has the variable degree γ=4, and the parity-check equation degree ρ=8. In the rest of the paper, this code will be denoted as i-RISC code. The numerical results will be presented for the maximum number of iterations $L_{max}=300$, if not specified otherwise.

**Figure 8:**
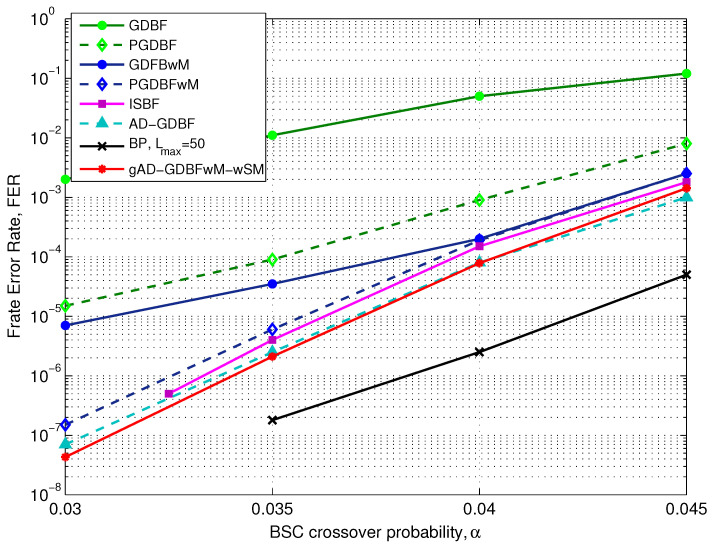
Performance of the various decoders, BSC, i-RISC code (1296, 648), γ=4 and ρ=8.

The FER performance for the i-RISC code and BSC is presented in Figure 8. Incorporating momentum into the energy function improves the GDBF decoder performance, and if it is combined with the randomness (as in the PGDBFwM), it can additionally reduce FER in the error floor region. The GDBF and the PGDBF algorithms are inferior to the GDBFwM and the PGDBFwM algorithms. The information storage bit flipping (ISBF) algorithm additionally improves performance compared to the PGDBFwM algorithm, but it also incorporates randomness in the decision process. On the other hand, the AD-GDBF algorithm achieves superior performance by concatenating completely deterministic decoders.

The performance of the gAD-GDBFwM-wSM algorithm for the BSC is presented for the case where the momentum vector and the decoder coefficients are optimally chosen. In the optimization process, three predefined momentum sets are used ($MS_{1}$, $MS_{2}$, and $MS_{3}$), the cases with $SM_{1}$ and $SM_{2}$, and both flag restart options were considered. In total, 12 combinations of the basic parameters were investigated, and the optimization of the momentum vector and the decoder coefficients was performed by using the GA for each combination. Finally, the combination with the highest fitness rate is chosen. The corresponding numerical results indicate that gAD-GDBFwM-wSM outperforms PGDBFwM, although it does not require statistically independent random sequences in the variable nodes. In addition, the gAD-GDBFwM-wSM algorithm achieves a similar performance as the AD-GDBF algorithm, as the effect of applying potential is less visible for the BSC.

**Figure 9:**
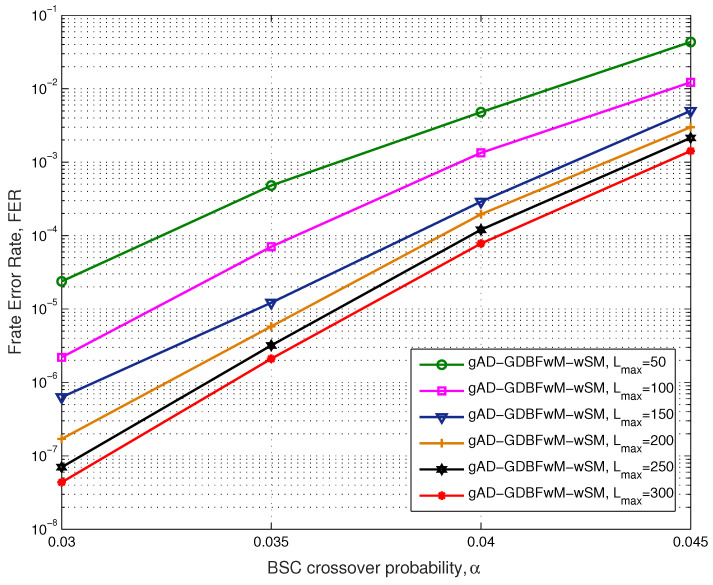
Performance of the various $L_{max}$, BSC, i-RISC code (1296, 648), γ=4 and ρ=8.

The performance of the gAD-GDBFwM-wSM algorithm for various parameter values $L_{max}$, for the BSC and the i-RISC code, can be seen in Figure 9. In this case, all component decoders terminate decoding after $L_{max,t}=50$ iterations, and the FER curves correspond to the case of various numbers of component decoders. For the second decoder, the fitness rate achieved is FR=1.90, which means that the decoder corrects 90 percent of the error patterns that were uncorrectable by the first component decoder. The difference between the FER curves for $L_{max}=50$ and $L_{max}=100$ is one decade at α=0.03. Generally, at the beginning of the cascade, the decoders greatly improve the performance compared to the case before applying that decoder. After the initial stages, performance improvement slows down, as the corresponding fitness rate has lower values (e.g., for the sixth decoder in the cascade, FR=1.40 is achieved, corresponding closely to 29 percent of corrected error patterns from the corresponding error log).

**Figure 10:**
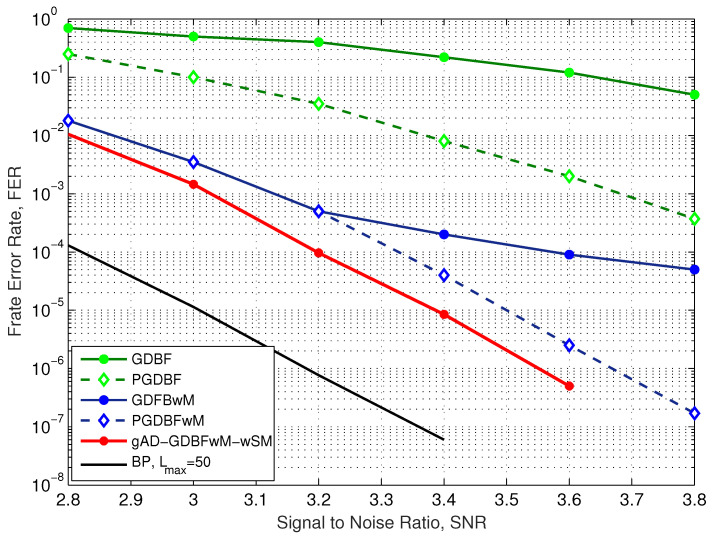
Performance of the various decoders, AWGN, i-RISC code (1296, 648), γ=4 and ρ=8.

In Figure 10, the performance is presented for the same code and the AWGN channel. For each combination of the predefined sets of the parameters ($MS_{1}$, $MS_{2}$, and $MS_{3}$), ($SM_{3}$, $SM_{4}$) and ($r_{flag}=0$, $r_{flag}=1$), the GA with 40 chromosomes and 40 generations is performed. For the decoders with the best fitness score, the FER(SNR) curves were plotted for $L_{max,i}=50$, i=1,2,...,6, and the decoder with a minimal value of FER is chosen. The error patterns that are correctable by using the chosen decoder are removed from the original database of the error patterns. After concatenation to the previously chosen decoders, the Monte Carlo simulation is performed for high SNR values to update a database of the error patterns. This database is used for the optimization of the next component decoder as input for the GA. It should be noticed that the database is updated after the new component decoder is chosen—as it can correct some patterns, these patterns are removed, and when the database becomes small, the new error patterns are added to provide a sufficient number of error patterns for an accurate optimization. In Figure 10, it is shown that our solution surpasses the existing PGDBFwM decoder by 0.1 dB, without using any probabilism in the decoding algorithm.

**Figure 11:**
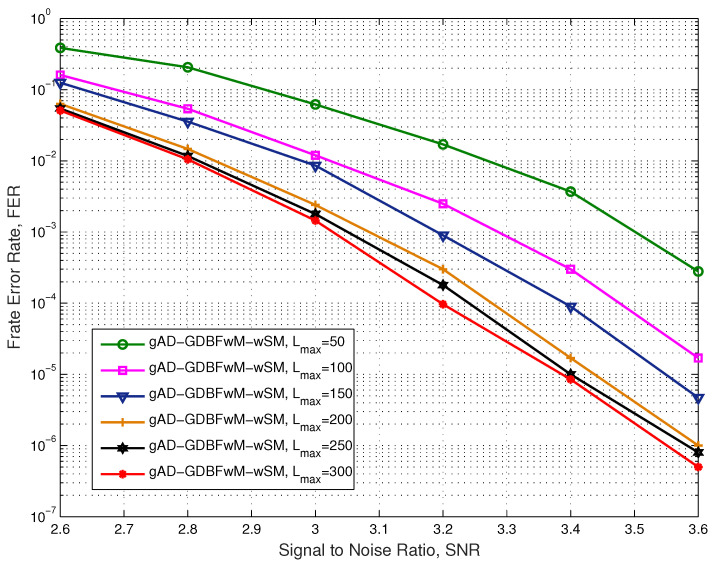
Performance of the various $L_{max}$, AWGN, i-RISC code (1296, 648), γ=4 and ρ=8.

The performance of the gAD-GDBFwM-wSM algorithm for various values of the parameter $L_{max}$ is also given for the case of the AWGN channel. The corresponding numerical results are presented in Figure 11. After adding the new decoders, the performance improvement starts steeply and then starts to slow down. The difference between two decoders in the chain and three decoders in the chain is larger than the difference between the four decoders in the chain and five decoders in the chain. This means that the fitness scores we obtain for the next decoders in the chain are decreasing. For example, for the second decoder in the chain, the fitness rate was FR=1.95, while for the fifth decoder in the chain, the fitness rate was FR=1.42.

Furthermore, FER steadily decreases by adding new decoders to the chain. This also has an impact on the adequate database of the error logs, since the error logs are produced for the higher SNR values (in those codewords, mostly TSs are present). However, with such an approach it becomes computationally complex to collect large numbers of error logs. Moreover, by observing Figure 10 and Figure 11, it can be seen that our solution with four decoders (200 iterations) exceeds the performance of the PGDBFwM decoder from [26] with 300 iterations. The significance of this result is related to the fact that the deterministic decoder outperforms the decoder with applied randomness for a smaller number of iterations.

### 4.3. IEEE 802.3an Code

**Figure 12:**
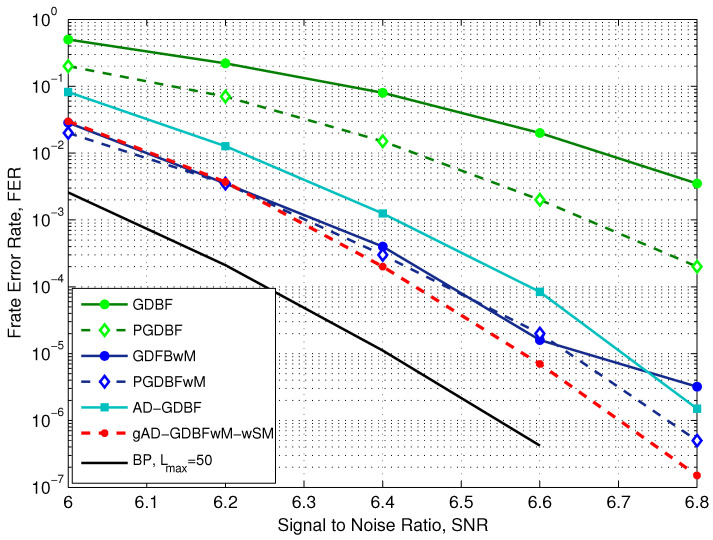
Performance of the various decoders, AWGN, IEEE 802.3an code (2048,1723), γ=6 and ρ=32.

Finally, the numerical results for the (2048,1723) code from the IEEE 802.3an standard [38] will be presented. This is a (γ,ρ)=(6,32) regular LDPC code with code rate R=0.8413. The length of the codewords is n=2048, with m=384 parity-check equations. The performance results of this code can be seen in Figure 12. In the remainder of the paper, this code will be called the IEEE 802.3an code. The numerical results will be presented for the maximum number of iterations $L_{max}=300$, if not specified otherwise.

Here, the learning process was performed only for the finite state machine with four states, i.e., $SM_{4}$. The experiments were performed using only one type of momentum set, $M_{1}$, because this momentum set tended to give the best results. The learning process also consisted of collecting several thousand error logs and learning from them several first decoders. When the error log was small, a new error log with several hundred error logs was obtained for higher SNR values, and the learning process continued with the new error log. The metric used here was only the number of corrected errors in the error log, i.e., the percentage of corrected error logs. This decoder is made out of six decoders, each with $L_{max,t}=50$ iterations.

Similarly to previous results for the AWGN channel and i-RISC code, it can be seen that the GDBF and the PGDBF algorithms have inferior performance when compared with the GDBFwM and the PGDBFwM algorithms. Using the combination of momentum and probabilism, the performance of the code can be significantly improved, especially in the error floor region. Performance can be further increased by using the gAD-GDBFwM-wSM principle. When compared to the PGDBF algorithm the proposed algorithm provides the additional improvement of almost 0.1 dB, as presented in Figure 12. We point out again that gAD-GDBFwM-wSM does not use any probabilistic principle, and there is no need for random generators in the implementation of the decoder. Comparison with the AD-GDBF algorithm, which uses the same parameters as the gAD-GDBFwM-wSM algorithm, is also given. It can be noticed that the gAD-GDBFwM-wSM algorithm proposed in this paper outperforms the other analyzed decoding algorithms.

## 5. Complexity Analysis

In this section, the complexity analysis will be described. Regarding the AD-GDBF algorithm, there is a complexity analysis for it in the work [31]. Our solution incorporates the state machine, but we show that the state machine does not add much complexity.

For every variable bit, there is a need to have additional memory resources regarding the state and the potential for every bit. Regarding potential, since the operations are performed in floating-point arithmetic, the solution needs one floating-point adder and one floating-point register for every bit, which will save the variable bit’s current potential.

For the state machine, every variable bit needs to have a memory item that shows in which state that variable bit is. This memory element can be used to store integer values in binary format. This can be performed with a simple register, the length of which depends on η and ζ. The first bit in the register can represent the sign, while the other bits can represent the value. Let us assume that the value of the state register represents the state of the variable bit, for example, if the variable bit is in the $S_{-,1}$ state, then in the register, the value −1 is written.

Depending on whether the variable bit is highlighted or not, similar operations are required. When the state changes, two comparisons are necessary, which compare the current state of the bit to 0 and check its sign. The comparison with zero can be conducted like an operation that checks if all the bits in the register are zeros (for example, multiple input NOR gate), and there is no need for a comparator there. To check the sign of the variable bit, we can only check the first bit in the register that shows in which state the variable bit is, so this operation does not require a comparator as well. After those operations, there is either a need to increment or decrement what is inside the state register, or to rewrite the state register. Increment and decrement operations are simple operations, while rewriting is more complex. However, when the register is rewritten, it is always written to the same value or only its sign changes, which makes the operation more complex friendly.

A comparator to compare the current value of the state of the variable bit with η or ζ depending on whether the variable bit is highlighted is needed. This needs to be carried out because the value in the register can be in some state that does not exist. This means that one comparator per bit is required.

In summary, for every variable bit, in addition to the AD-GDBFwM, there is a need for as follows:

- One floating-point register;
- One floating-point adder;
- One register for the state of that variable;
- One comparator.

## 6. Conclusions

In this paper, we have proposed the gAD-GDBFwM-wSM algorithm where the concatenation of several decoders with momentum is combined with the concept of variable node potential. The update of the potential has been realized with the deterministic finite state machine, where the state transitions are dictated by the past highlighting activity of the variable node. We have illustrated the proposed update mechanism in the case of a rather complex trapping set. We have also proposed a general learnable framework for the optimization of the decoder parameters. The optimization by using the GA has been described in detail, and the corresponding numerical results have been given.

It can be seen that the proposed algorithm achieves superior performances when compared to the previously proposed GDBF-based algorithms. The gAD-GDBFwM-wSM algorithm outperforms its probabilistic counterparts (e.g., PGDBF, PGDBFwM, TRGDBF, ISBF) for the same number of iterations. As no randomness was used in our algorithm, there is no need to incorporate random generators during the implementation of the decoder. For short codes, the proposed algorithm even outperforms the state-of-the-art BP algorithm. The proposed framework is applicable to any regular LDPC code, both for the BSC and the AWGN channel.

The limitations of the gAD-GDBFwM-wSM algorithm are mostly related to the time-consuming learning process. In order to find the decoder with the optimal parameters, the error logs with a few thousand error patterns have to be generated and regularly updated before the design of every particular component decoder. Even if the GA is applied for the optimization, it is necessary to run it for various combinations of the system parameters. However, it is important to notice that the optimization can be performed offline. Once determined, the optimal parameters of the gAD-GDBFwM-wSM decoder enable high reliability of the transmission. In our future work, we will concentrate on developing faster and more effective optimization using the GA or its competitive algorithms.

Furthermore, the GDBF algorithm has oscillatory behavior when TSs are present, as shown in our example in Section 3. In our examples and simulations, we did not notice that gAD-GDBFwM-wSM has oscillatory behavior. The exact analysis of possible oscillatory behavior for the gAD-GDBFwM-wSM algorithm is a complex task and will be the topic of our future work.

## Figures and Tables

**Table 1:** Table of used symbols and their meaning.

Symbol	Meaning
*n*	codeword length
*k*	number of information bits
*R*	code rate
$H_{m\times n}$	parity-check matrix
$h_{j,i}$	element of a parity-check matrix
$v_{i}$	variable node associated to *i*-th column
$c_{j}$	parity-check equation related to the *j*-th row
$P(v_{i})$	set of indices for $v_{i}$
$Q(c_{j})$	set of indices for $c_{j}$
γ	regular LDPC code, degree of $v_{i}$
ρ	regular LDPC code, degree of $c_{j}$
x	transmitted codeword
y	received codeword
$\hat{x}$	estimated codeword
$L_{max,t}$	maximum no. iterations for *t*-th decoder
$L_{max}$	maximum no. iterations for chained decoder
$r_{flag}^{\left(t\right)}$	restart flag of the *t*-th decoder
$E_{i}^{\left(\ell \right)}$	Energy function for *i*-th VN in the *ℓ*-th iteration
$w_{1}$, $w_{2}$	learnable weights
$m_{\mu_{i}}$	value of momentum for variable *i*
$\mu_{i}$	momentum state of the *i*-th variable
m	momentum vector
$L^{\prime }$	number of values in momentum vector
*I*	maximum value in momentum vector
$F^{\left(\ell \right)}$	set of highlighted variables in *ℓ*-th iteration
δ	margin (threshold) parameter
$r_{i}^{\left(\ell \right)}$	potential for variable $v_{i}$ in *ℓ*-th iteration
θ	value for θ rule
$r_{in}^{\left(t\right)}$	vector of potentials for *t*-th decoder
$\mu_{in}^{\left(t\right)}$	vector of momentum states for *t*-th decoder
$P_{in}^{\left(t\right)}$	vector of states for finite state machine
S	finite state machine
*S*	state in the finite state machine
*s*	value of state in the finite state machine
$S_{+}$	set of + states in the finite state machine
η	number of states in $S_{+}$
$S_{-}$	set of − states in the finite state machine
ζ	number of states in $S_{-}$
$S_{0}$	neutral state
$S_{start}$	starting state
$S_{t}$	function which converts finite state machines

**Table 2:** Momentum set parameters used in GA optimization.

Momentum Set Name	$L^{\prime }$	*I*
$MS_{1}$	2	3
$MS_{2}$	3	2
$MS_{3}$	4	2
$MS_{4}$	3	3

**Table 3:** Finite state machine, dimension parameters used.

Finite State Machine Name	No. State |S|	ζ	η
${SM}_{1}$	5	2	2
${SM}_{2}$	3	1	1
${SM}_{3}$	2	1	0
${SM}_{4}$	4	2	1

## Data Availability

The original data presented in the study are openly available in web page https://github.com/jovan94/MDPI_StateMachineForPotentials (accessed on 1 December 2024).
